# Notes on the Cultivation of Two Mixotrophic *Dinophysis* Species and Their Ciliate Prey *Mesodinium rubrum*

**DOI:** 10.3390/toxins10120505

**Published:** 2018-12-01

**Authors:** Jorge Hernández-Urcera, Pilar Rial, María García-Portela, Patricia Lourés, Jane Kilcoyne, Francisco Rodríguez, Amelia Fernández-Villamarín, Beatriz Reguera

**Affiliations:** 1 Microalgas Nocivas, Centro Oceanográfico de Vigo, IEO, Subida a Radio Faro 50, 36390 Vigo, Spain; jurcera@iim.csic.es (J.H.-U.); pilar.rial@ieo.es (P.R.); maria.garcia@ieo.es (M.G.-P.); patricia.loures@ieo.es (P.L.); francisco.rodriguez@ieo.es (F.R.); amelia.fernandez@ieo.es (A.F.-V.); 2 Biotoxin Chemistry, Marine Institute, Rinville, Oranmore, Co. Galway H91 R673, Ireland; jane.kilcoyne@marine.ie

**Keywords:** *Dinophysis*, *Mesodinium*, cryptophytes, predator-prey preferences, Diarrhetic Shellfish Toxins (DST), pectenotoxins (PTXs), mixotrophic cultures, mass culture conditions

## Abstract

Kleptoplastic mixotrophic species of the genus *Dinophysis* are cultured by feeding with the ciliate *Mesodinium rubrum*, itself a kleptoplastic mixotroph, that in turn feeds on cryptophytes of the *Teleaulax*/*Plagioselmis*/*Geminigera* (TPG) clade. Optimal culture media for phototrophic growth of *D. acuminata* and *D. acuta* from the Galician Rías (northwest Spain) and culture media and cryptophyte prey for *M.* *rubrum* from Huelva (southwest Spain) used to feed *Dinophysis*, were investigated. Phototrophic growth rates and yields were maximal when *D. acuminata* and *D. acuta* were grown in ammonia-containing K(-Si) medium versus f/2(-Si) or L1(-Si) media. *Dinophysis acuminata* cultures were scaled up to 18 L in a photobioreactor. Large differences in cell toxin quota were observed in the same *Dinophysis* strains under different experimental conditions. Yields and duration of exponential growth were maximal for *M. rubrum* from Huelva when fed *Teleaulax amphioxeia* from the same region, versus *T. amphioxeia* from the Galician Rías or *T. minuta* and *Plagioselmis prolonga*. Limitations for mass cultivation of northern *Dinophysis* strains with southern *M. rubrum* were overcome using more favorable (1:20) *Dinophysis*: *Mesodinium* ratios. These subtleties highlight the ciliate strain-specific response to prey and its importance to mass production of *M. rubrum* and *Dinophysis* cultures.

## 1. Introduction

Several mixotrophic species of the genus *Dinophysis* produce one or two groups of lipophilic toxins: (i) okadaic acid (OA) and its derivatives, the dinophysistoxins (DTXs), and (ii) pectenotoxins (PTXs) [1,2]. The OA and DTXs, known as Diarrhetic Shellfish Poisoning (DSP) toxins, are acid polyethers that inhibit protein phosphatase and have diarrheogenic effects in mammals. The PTXs are polyether lactones, some of which are hepatotoxic to mice by intraperitoneal injection [3]. Their toxicity has been questioned, since they do not appear to be toxic when ingested orally [4]. Nevertheless, they are still subject to regulation in the European Union (EU). The two groups of toxins, OA related toxins and PTXs, can now be analyzed with independent analytical methods, which have led the EU to regulate them separately [5].

DSP toxins pose a threat to public health, and together with PTXs, cause considerable losses to the shellfish industry globally [6,7]. Harvest closures are enforced when toxin levels exceed local regulatory limits (RL). *Dinophysis* blooms, in particular those of *D. acuminata* and *D. acuta*, are persistent in western Iberia (Spain and Portugal). Contamination of shellfish with *Dinophysis* toxins above the RL can last up to nine months in the most affected aquaculture sites [8,9]. Harmful algal blooms (HABs), in particular *Dinophysis* blooms, cannot be eliminated, therefore, more detailed knowledge of the conditions affecting *Dinophysis* growth and toxin production is crucial to improve risk forecasting. Forecasts can help the shellfish industry schedule harvest plans and help mitigate the deleterious impacts of such blooms.

Protection of public health and seafood safety control require the implementation of costly monitoring systems; these include frequent toxin analyses of all commercially exploited shellfish species with sophisticated analytical instruments, such as liquid chromatography coupled to tandem mass spectrometry (LC–MS/MS) [8,9]. These chemical methods require pure certified toxin standards for the analyses, which are difficult to obtain or are yet to be developed. Successful cultivation of *Dinophysis* in the laboratory is instrumental for addressing these shortfalls. Of particular importance is the optimization of mass production of *Dinophysis* to allow isolation and purification of toxins. Further, some *Dinophysis* toxins may have a wide spectrum of applications. For example, PTX2 has been found to cause a selective apoptosis of carcinogenic cells [10,11], and currently, protocols for the mass production of *D. acuminata* in Korea to obtain PTX2 for the pharmaceutical industry have been patented [12].

For years, the establishment of *Dinophysis* cultures challenged microalgal physiologists. *Dinophysis* species were found to bear unusual plastids containing pigments—phycoerythrins—and a structure similar to those of cryptophyte microflagellates [13]. Attempts to grow them with conventional culture media used for dinoflagellates, with addition of dissolved organic matter or even with bacteria were unsuccessful [14]. The observation of ciliate remains in the digestive vacuoles of *D. acuminata* and *D. norvegica* confirmed their mixotrophic nature [15]. The next breakthroughs came with the application of molecular tools. DNA sequences of the plastid SSU rRNA gene of *Dinophysis* were found to coincide with those from living cryptophytes closely related to *Geminigera cryophila* [16]. A correlation between *Dinophysis* and cryptophyte cell densities in the field, estimated with molecular probes, was found [17], but attempts to grow *Dinophysis* directly fed with cryptophytes were unsuccessful [18]. 

Further studies showed that partial sequences of the plastid *psbA* gene and the ribosomal 16S rRNA gene from *Dinophysis* species were identical to the same loci in living cryptophyte *Teleaulax amphioxeia.* These findings raised the suspicion that *Dinophysis* plastids were stolen plastids (kleptoplastids). The key question was whether *Dinophysis* acquired these kleptoplastids through an intermediate organism [19]. A few years earlier, the first culture of the phototrophic ciliate *M. rubrum* to feed the cryptophyte *Geminigera cryophyla* was achieved [20]; its feeding behavior taking up crytophytes (*T. amphioxeia)* through an oral cavity was described [21]. Finally, the first successful culture of *D. acuminata* using the ciliate *M. rubrum*, grown with *T. amphioxeia* as prey was established. *Dinophysis* was found to feed on *M. rubrum* by myzocytosis, a type of phagotrophy where the predator pierces the prey with a feeding peduncle and sucks its content. After the feeding process, *Dinophysis* appeared full of digestive vacuoles, but the prey plastids were retained and used as kleptoplastids [22]. 

Since then, cultures of several *Dinophysis* species—*D. acuta* [23], *D. caudata* [24], *D. fortii* [25], *D. infundibulu*s [26], *D. sacculus* [27], and *D. tripos* [28]—have been established via this three-species chain of serial kleptoplastidy, i.e., cryptophyte plastid acquisition from the TPG clade (*Teleaulax*/*Plagioselmis*/*Geminigera)* to *M. rubrum*, which in turn provides plastids to *Dinophysis*. Small-volume cultures, ranging from a few mL in multiwell plates to Erlenmeyer flasks of 250 mL, based on the same kind of mixotrophic nutrition, were set up to carry out physiological, toxinological and genetic studies. These *Dinophysis* species were cultivated with the ciliate *M. rubrum* fed two cryptophytes belonging to the TPG clade (i.e., *Teleaulax amphioxeia* or *Geminigera cryophila*) using full or diluted f/2 [29] or L1 medium [30]. 

The mass production of *D. acuminata* to obtain pectenotoxins, including production of the ciliate *M. rubrum* and cryptophyte of the genus *Teleaulax* to feed *Dinophysis*, has been addressed and the results patented. Nevertheless, the exact details of the *Teleaulax* species used to feed *M. rubrum* were not provided in the patent description [12]. Maintaining a balance between the three species of the ‘cryptophyte–ciliate–dinoflagellate’ food chain is difficult, because each has different requirements. These requirements range from the purely autotrophic *T. amphioxeia*, to mixotrophic *M. rubrum* and *Dinophysis* species, which require light and live prey for sustained growth [31,32,33]. Nevertheless, *M. rubrum* only needs to ingest 1–2% of its daily carbon intake from its prey to attain maximum growth, whereas *Dinophysis* species require ~50% for the same purpose [31]. Both *M. rubrum* and *Dinophysis* species can survive for months in the light without food, and their light preferences are different from the cryptophytes [33].

This work is a compilation of original observations and problems frequently faced in the maintenance and optimization of *Dinophysis*, *M. rubrum*, and cryptophyte cultures. Observations are from strain maintenance in the culture collection and experiments carried out at the IEO-Vigo laboratory, Nantes, France [34] and Naples, Italy [35] where the same *Dinophysis* and *M. rubrum* strains were used. The objectives of this work were: (i) to optimize culture medium for *Dinophysis* (*D. acuminata* and *D. acuta*) from the Galician Rías (northwest Spain), the ciliate prey *M. rubrum* from Huelva (southwest Spain) and different cryptophyte prey species; (ii) to estimate growth and yields of *M. rubrum* grown with cryptophyte species different from *T. amphioxeia*; and (iii) to optimize cryptophyte prey for *M. rubrum* for maximal *Dinophysis* growth and yield.

## 2. Results

### 2.1. Optimizing Culture Medium for Phototrophic Growth of *D. acuminata* and *D. acuta*

The objective of this experiment was to test which of the three culture media (f/2 [29], L1 [30] and K [36]) was best for the phototrophic growth (no prey added) of *D. acuminata* (VGO1391) and *D. acuta* (VGO1065) from the Galician Rías (northwest Spain), and if the best medium for *Dinophysis* growth coincided with the best for their ciliate prey *M. rubrum* (AND-A071) from Huelva (southwest Spain). 

**Figure 1:**
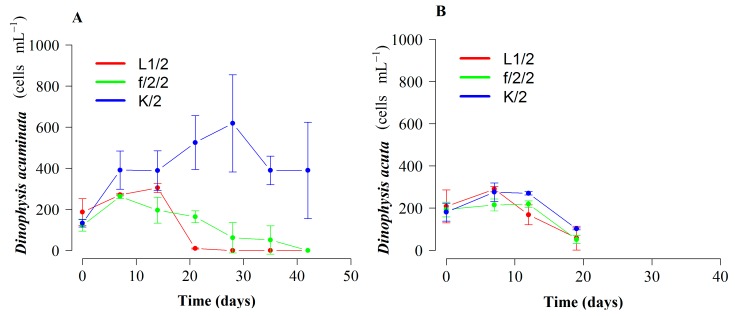
Phototrophic growth (prey-depleted) of *Dinophysis* species with three different diluted (1:2), Si free culture media (L1, f/2 and K). (**A**) *D*. *acuminata* (VGO1391) and (**B**) *D*. *acuta* (VGO1065). Bars represent standard error.

*Dinophysis acuminata* cell densities increased moderately the first 7–10 days with the three treatments. From day 14 onwards, cultures grown with diluted (1:2) f/2 and L1 media started to decline. Cultures with diluted (1:2) K(-Si) medium showed 7-d stationary phase (day 7 to 14) followed by exponential growth (µ = 0.15 d^−1^) until day 28, reaching 619 cells mL^−1^. By day 42, cell density in cultures with K(-Si) medium was 390 cells mL^−1^ (mean), whereas no cells were observed in the cultures with diluted f/2 and L1 media (Figure 1A). Therefore, duration of exponential growth was 14 d longer and the final yield was significantly higher (*p* = 1.3 × 10^−6^) in *D. acuminata* cultures with K(-Si) medium. 

**Figure 2:**
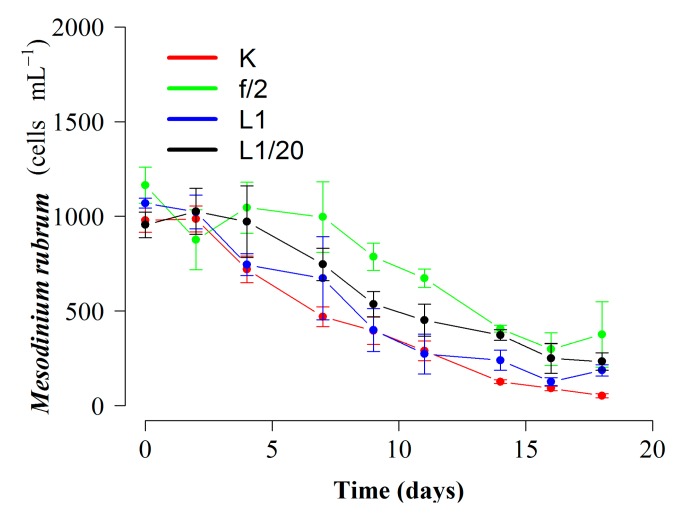
Phototrophic growth (no cryptophyte prey) of *M. rubrum* (AND-A071) cultures (previously fed with *T. minuta*, CR8EHU) with different Si free enrichment media (K, f/2, L1 and diluted, 1:20, L1). Bars represent standard error.

Regarding *D. acuta*, maximal growth rate—also obtained with K(-Si) medium—was extremely low (µ = 0.06 d^−1^) and positive growth lasted seven days only. Differences between treatments were not statistically significant (*p* = 0.67) (Figure 1B). Phototrophic (no cryptophyte prey added) growth rates of the prey, *M. rubrum*, with the three different culture media in 250 mL at 15 °C were similar for the duration of the experiment, but the initiation of the exponential decline was later (day 7) and the final yield maximal (*p* < 0.02) with f/2(-Si) medium (Figure 2).

### 2.2. Growth and Cell Toxin Quota in 4 L Mixotrophic Cultures of *D. acuminata* and *D. acuta*

**Figure 3:**
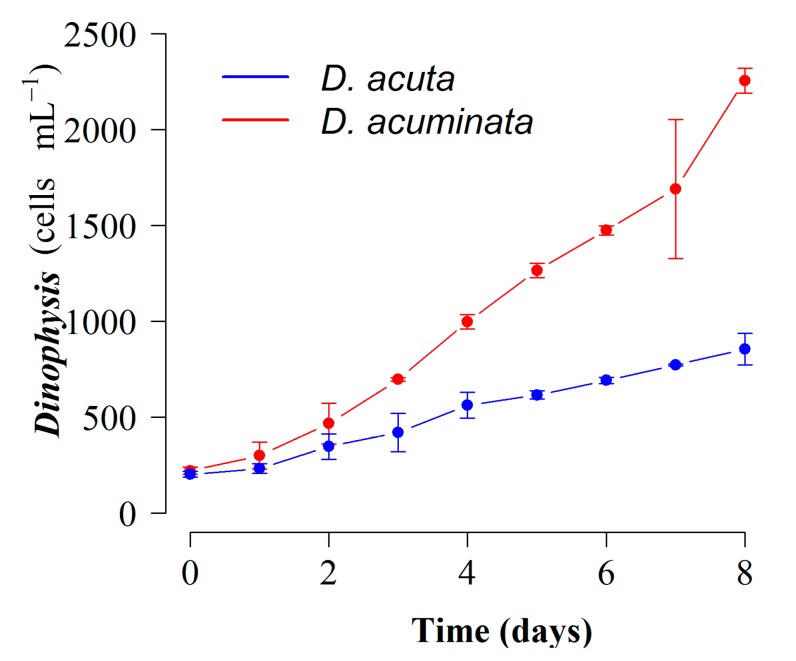
Growth curves of mixotrophic cultures of *D. acuminata* (VGO1391) and *D. acuta* (VGO1065) (fed *M. rubrum*, AND-A071) grown with *P. prolonga*, CR10EHU) with K(-Si) medium in 4 L flasks at 19 °C and a 16L:8D cycle. Bars represent standard error (*n* = 9).

Scaled-up (4 L) mixotrophic cultures of *D. acuminata* and *D. acuta* with full K(-Si) medium and addition of *M. rubrum* (AND-A071) prey showed maximal growth rates of µ = 0.33 d^−1^ and 0.26 d^−1^ respectively in short-term experiments at 19 °C and a 16:8 light:dark cycle. Final yields by day 8 were 2287 cells mL^−1^ for *D. acuminata* and 883 cells mL^−1^ for *D. acuta* (Figure 3). Toxin contents were 9.9 pg OA cell^−1^ in *D. acuminata* and 7.7 pg OA + 2.9 pg DTX2 + 8.2 pg PTX2 cell^−1^ in *D. acuta* (Table 1).

### 2.3. Optimization of *M. rubrum* Prey

**Figure 4:**
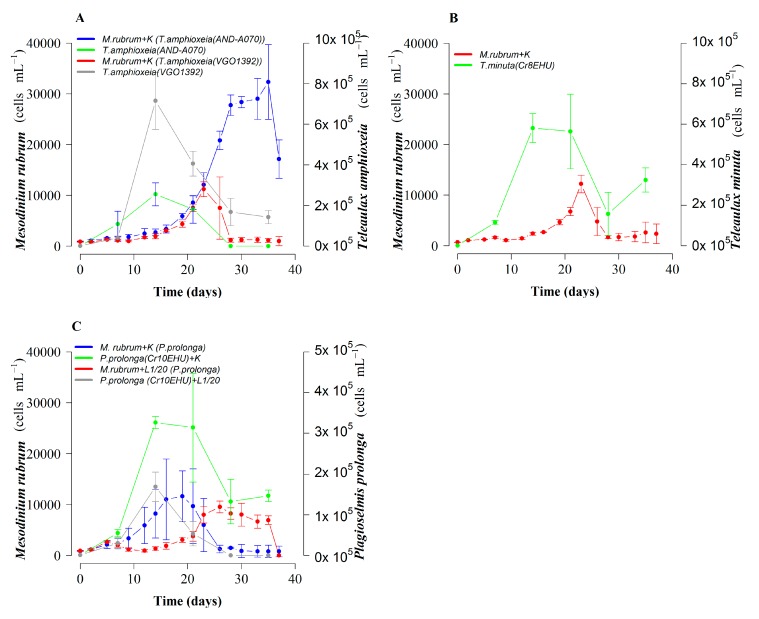
Growth of *M. rubrum* (AND-A071) fed three cryptophyte species with K(-Si) medium: (**A**) *T. amphioxeia* strains from Vigo (VGO1392) and Huelva (AND-A070); (**B**) *T. minuta* (Cr8EHU), and (**C**) *P. prolonga* (Cr10EHU) from the Basque Country, north Spain, with K(-Si) and L1/20 (-Si) culture media. Bars represent standard error.

Mixotrophic growth of *M. rubrum* from Huelva (southwest Spain), fed with different species of the TPG clade from different Spanish regions was tested. Growth curves of *M. rubrum*, previously grown with *P. prolonga* in K(-Si) medium, showed a lag phase of more than 10 days in mixotrophic cultures while being fed two different strains of cryptophye *T. amphioxeia*: strain AND-A070 from Huelva (southwest Spain) and strain VGO1392 from the Galician Rías (northwest Spain) respectively (Figure 4A). This was followed by a moderate (µ = 0.18 d^−1^ between day 16 and 28) exponential growth until day 35, and an abrupt decline after reaching the maximal yield (30–50 × 10^3^ cells mL^−1^) in cultures fed *T. amphioxeia*, strain AND-A070, i.e., from the same area as the ciliate. Final yields in *M. rubrum* cultures fed the same cryptophyte species, *T. amphioxeia* strain VGO1392, but from northwest Spain, were three times smaller. Growth rates with this strain were lower and comparable with those observed in cultures fed with *T. minuta* (*p* = 0.02) (Figure 4B). In fact, the growth curves of *M. rubrum* cultures fed *T. amphioxeia* from northwest Spain and *T. minuta* showed very similar patterns and both reached the maximal yield after three weeks. Cultures fed *P. prolonga* with K(-Si) medium showed a moderate growth (µ = 0.17 d^−1^) between day 9 and 16, and entered a plateau phase on day 19, followed by a fast decline (Figure 4C). Cultures of *M. rubrum* with *P. prolonga* in diluted (1:20) L1(-Si) medium, used as an internal control, exhibited a maximal yield slightly lower than those fed the same cryptophyte with K(-Si) medium, but growth rate over the first two weeks was very low and it took an additional week to reach the maximal yield.

### 2.4. Optimal Cryptophyte Prey for *M. rubrum* and *Dinophysis:Mesodinium* (D:M) Ratio for Highest *Dinophysis* Growth and Survival

**Figure 5:**
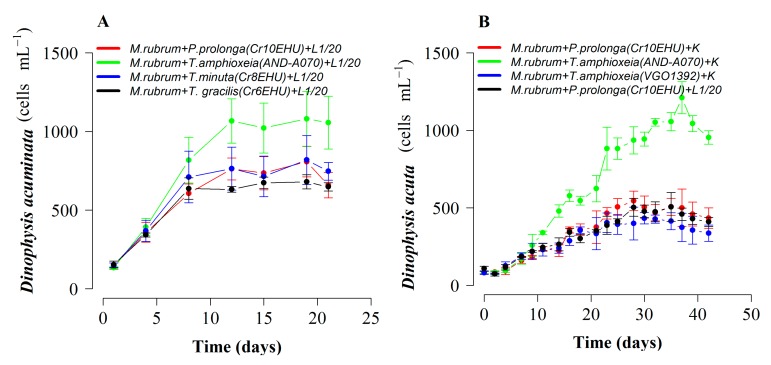
Growth of *Dinophysis* species with *M. rubrum* (AND-A071) fed different crytptophyte species. (**A**) *D. acuminata* with *M. rubrum* fed *T.amphioxeia* (AND-A070), *T. minuta* (Cr8EHU*)*, *T. gracilis* (Cr6EHU), and *P. prolonga* (Cr10EHU). (**B**) *D. acuta* with *M. rubrum* fed two crytptophyte species: *T. amphioxeia* (AND-A070, VGO1392) and *P. prolonga* (Cr10EHU). Bars represent standard error.

*Dinophysis acuminata* was able to grow with *M. rubrum* fed four cryptophyte species growing in diluted (1:20) L1 medium. Growth rate, and in particular final yield of *D. acuminata* achieved with *M. rubrum* fed *T. amphioxeia*, strain AND-A070 (µ = 0.36 d^−1^; 1.11 × 10^3^ cells mL^−1^), were higher than those with *T. minuta* (µ = 0.33 d^−1^; 0.82 × 10^3^ cells mL^−1^), *T. gracilis* (µ = 0.27 d^−1^; 0.68 × 10^3^ cells mL^−1^), and *P. prolonga* (µ = 0.30 d^−1^; 0.81 × 10^3^ cells mL^−1^) (*p* = 0.016) (Figure 5A). Differences were more marked in the case of *D. acuta* grown with *M. rubrum* fed *T. amphioxeia* (AND-070). These cultures showed four weeks of sustained exponential growth phase (µ = 0.2 d^−1^), from day 10 to 38, and a final yield of 1.21 × 10^3^ cells mL^−1^. In contrast, shorter exponential growth phase and about half the final yield were achieved in cultures with the same species fed: *T. amphioxeia (*VGO1392) (µ = 0.26 d^−1^; yield: 0.43 × 10^3^ cells mL^−1^), *P. prolonga* (CR10EHU) grown with full K(-Si) medium (µ = 0.23 d^−1^; 0.54 × 10^3^ cells mL^−1^) and *P. prolonga* with diluted (1:20) L1 (µ = 0.22 d^−1^; 0.5 × 10^3^ cells mL^−1^) (*p* = 1.3 × 10^−15^) (Figure 5B). Thus, a longer exponential phase and a 2-fold higher yield were obtained in *D. acuta* cultures with *M. rubrum* fed the *T. amphioxeia* (AND-A070) from the same location. Results from *D. acuta* cultures with *M. rubrum* fed *P*. *prolonga* were about the same whether the ciliate + cryptophyte had been growing with K(-Si) medium or with diluted (1:20) L1(-Si) medium.

**Figure 6:**
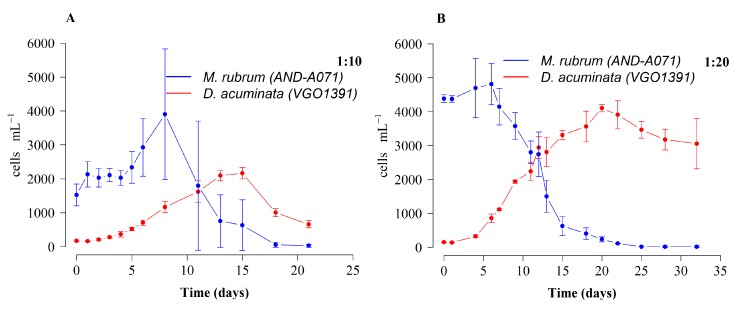
Growth of *D. acuminata* (VGO1391) fed *M. rubrum* (AND-A071) grown with *T. minuta* (CR8EHU) and K(-Si) medium with different predator:prey ratio. (**A**) *D. acuminata:M.rubrum* 1:10 and (**B**) *D. acuminata:M. rubrum* 1:20. Bars represent standard error.

*Dinophysis acuminata* cultures fed *M. rubrum* (which in turn was fed *T. minuta*) with a 1:20 D:M ratio, showed a higher growth rate (µ = 0.28 d^−1^), a two-fold higher final yield (4000 cells mL^−1^), and seven more days of sustained exponential growth as compared with the same strain of *D. acuminata* (µ = 0.21 d^−1^) fed with a 1:10 D:M ratio (Figure 6).

### 2.5. Mass Cultivation and Total Toxin Yield of *Dinophysis* in 30 L Photobioreactors

**Figure 7:**
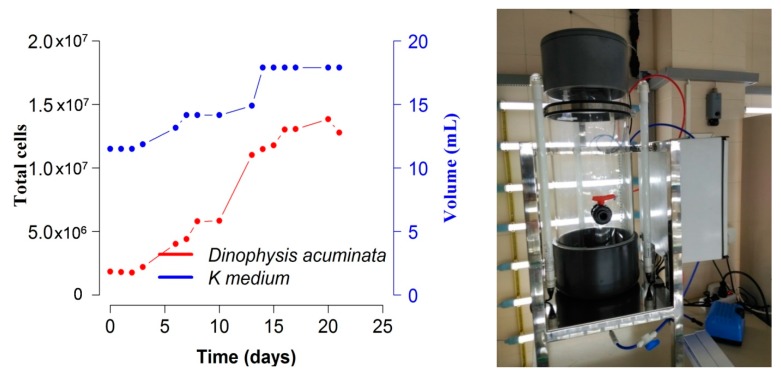
Cultures of *D. acuminata* with *M. rubrum* fed *T. amphioxeia* in a 30 L photobioreactor (right hand picture) with K(-Si) medium at 18 °C. Blue line indicates changes in volume in the photobioreactor.

A final yield of 0.77 × 10^3^ cells mL^−1^ of *D. acuminata* was obtained after 20 d of culture with a final volume of 18 L. Maximal growth rate achieved, between days 6 and 8 was 0.28 d^−1^ (Figure 7). LC–MS/MS analysis of total toxins (particulate and dissolved) adsorbed with the Diaion® resins revealed a content of 22.3 ng OA mL^−1^, corresponding to 770 cell mL^−1^ of *D. acuminata* and the extracellular toxins released in the culture medium.

### 2.6. *Dinophysis* Vertical Distribution in the Culture Vessels

In small- (≤250 mL) and medium- (several L) volume cultures of *Dinophysis*, cells were usually distributed in the bottom of the container. When depleted prey was replenished, observation of the cultures with the inverted microscope showed that *Dinophysis* cells swam upwards to catch *M. rubrum*. Otherwise, in samples collected after a gentle but thorough shaking of the containers to estimate cell densities, it was common to observe prey cells still attached to *Dinophysis* through a feeding peduncle. In contrast, in the large volume (up to 25 L) cultures in the bioreactor, *Dinophysis* cells could be observed in the water column forming patches above the level of the black plastic ring that protects the base of the metacrylate bioreactor (Figure 7) and in the air–water interface.

### 2.7. Nanoflagellate Contamination

Not infrequently, mixotrophic cultures of *Dinophysis* appeared contaminated with a tiny (~10 µm) nanoflagellate. Its growth became out of control and smothered *M. rubrum* cultures when either full f/2 or L1 media were used. The use of diluted (1:20) L1(-Si) medium, often used to control overgrowth of the cryptophyte in mixotrophic cultures of *M. rubrum*, proved to be effective in controlling the contaminating nanoflagellate. Mass cultures of *Dinophysis* became contaminated sometimes with it. In those cases, *Dinophysis* toxins from the cells and culture medium were cropped with the adsorbing Diaion® resins before the culture started to decline. The contaminating nanoflagellate was established in culture, sequenced, and identified as an undetermined chrysophyte species of the genus *Ochromonas*.

### 2.8. Sequencing and Phylogenetic Analysis

**Figure 8:**
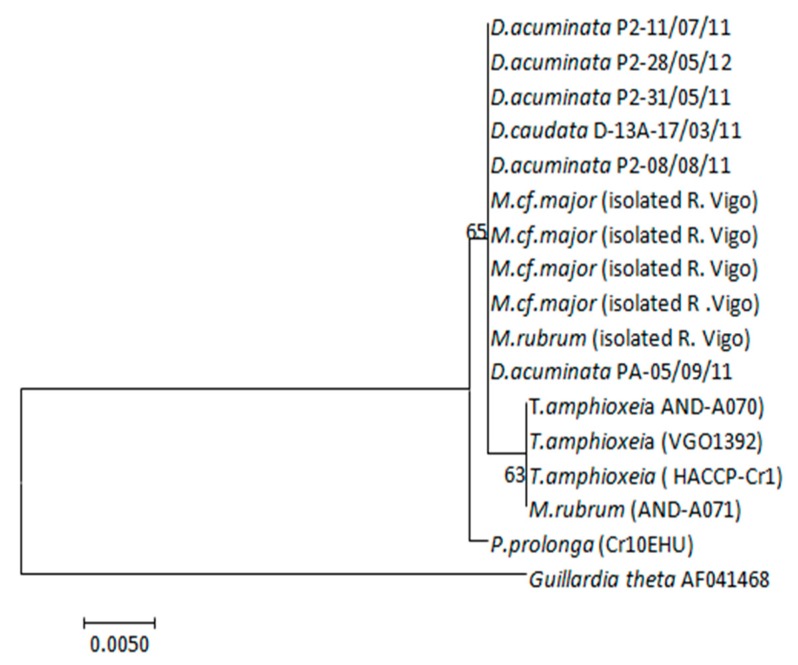
Maximum-likelihood (ML) tree inferred from partial plastid 23S rDNA sequences of *D. acuminata*, *D. caudata*, *T. amphioxeia*, *M. rubrum*, *P. prolonga*, and *M. cf. major*. Support at internal nodes is based on bootstrap values of ML methods with 1000 resamplings. *Guillardia theta* was added as outgroup to root the tree. Scale bar indicates number of substitutions per site.

Partial plastid 23S rDNA sequences (373 base pairs, bp) of *T. amphioxeia* (strains AND-A070 from Huelva and VGO1392 from Vigo) and *P. prolonga* (CR10EHU, north Spain) cultures, *M. rubrum* (cultivated strains AND-A071 from Huelva and isolated field specimens from Vigo) and *Mesodinium major* and *Dinophysis* isolated specimens from the Galician Rías (see isolation dates in Table 2) were almost identical (Table 2). In fact, a single base pair (bp) difference (out of the 373) was found in the amplified region between the plastids of *M. rubrum* and *T. amphioxeia* from Huelva versus those from the Galician specimens (*M. rubrum*, *M*. *major*, and *T. amphioxeia*). *Plagioselmis prolonga*, from the Basque country, differed in one additional base pair from these organisms (Figure 8).

## 3. Discussion

### 3.1. K(-Si) Medium Best for *Dinophysis* Growth

The most recent research with *Dinophysis* cultures has been carried out using full or diluted f/2 medium [29] for the dinoflagellate, the ciliate, and the cryptophytes, and in a few cases, L1 medium [30]. These enrichment media have only nitrate as a nitrogen source. Earlier studies showed that inorganic nutrients (nitrates and phosphates) provided in the culture medium were not used by *D. acuminata* and led to the conclusion that this species fulfilled its nitrogenous and phosphorous needs from ingested ciliate prey [37]. Incubation of field populations during a *D. acuminata* bloom in the Benguela upwelling system, South Africa, with radiolabeled (N^15^) nitrogenous compounds had shown this species had a great affinity for regenerated N compounds, such as ammonium and urea [38]. Culture incubations confirmed *D. acuminata* preference for ammonia, urea, and other organic forms of nitrogen rather than nitrate (new production) [39]. Recent studies found similar results and an apparent inability to use nitrate in cultures of the *D. acuminata* and *D. acuta* strains used in the present study [40]. These results led us to test K(-Si) medium [36] for *Dinophysis* cultivation, because it is the only one, among the commonly used culture media for dinoflagellates, which includes ammonium in the form of ammonium chloride (NH_4_Cl) as a nitrogen source. This better explains the results obtained, in terms of growth rate and yield, in phototrophic cultures of *D. acuminata* when using this culture medium (Figure 1A). 

Growth rate (µ < 0.1 d^−1^) and yields (<400 cells mL^−1^) obtained in phototrophic cultures of *D. acuta* grown with K(-Si) medium (experiment 1) were very poor (maximum of one doubling of the population), and slightly higher than with f/2 and L1 media (Figure 1B). This strain of *D. acuta* (VGO1065) had shown lower division rates than *D. acuminata* in all previous studies [40]. But the same strain of *D. acuta* showed a much higher growth (µ = 0.26 d^−1^) in the second experiment, where ciliate prey was supplied (mixotrophic growth), temperature (19 °C) was 4 °C higher, and the cycle had 4 h additional light. There is not enough information available to reach definitive conclusions, but a preliminary interpretation is that *D. acuta*, a late summer species in Western Europe, grows better with higher temperatures. Additionally, it can be speculated that heterotrophic growth is more important in *D. acuta* than in *D. acuminata.* This last hypothesis agrees with results obtained by García-Portela et al. [33], who found *D. acuta* had a much higher survival (30% of the initial population) than *D. acuminata* (10%) after four weeks in dark conditions. This hypothesis implies that *D. acuta* will suffer more from lack of prey than *D. acuminata*. In addition, the same D:M ratio was provided, in all the experiments, to the two species, although *D. acuta* is three times larger in terms of biovolume [33].

To date, culture experiments have grown both the ciliate *M. rubrum* and its cryptophyte prey in f/2 medium [41,42,43,44,45,46]. In our work, cellular yields of *M. rubrum* were similar with K and f/2 media. The three culture media tested here (f/2(-Si), L1(-Si), and K(-Si)) have extremely high (880 µM) concentrations of nitrates. This excess of inorganic nutrients favors the autotrophic cryptophytes (e.g., *Teleaulax*), with a much higher growth rate than *M. rubrum* and *Dinophysis*. A frequent problem is that *Teleaulax* overgrows and even smothers *M. rubrum* cultures. This problem is exacerbated when the cryptophyte species/strain chosen is not the best ciliate’s prey and grazing rates are lower [42,43]. This explains the common use of diluted f/2 medium in *Dinophysis* and *M. rubrum* culture experiments [33,34,41] in an attempt to prevent *Teleaulax* taking over.

In summary, K(-Si) is the best enrichment medium for growing *Dinophysis*, whether in small containers or in medium-scale volumes in photobioreactors. The use of diluted (1:20) L1(-Si) medium seems a good choice for long-term maintenance of *M. rubrum* and *Dinophysis* cultures. Despite showing lower cell densities than with full strength media, *Dinophysis* cells continue to grow and the risk of proliferation of *Ochromonas* and other contaminating small flagellates is reduced.

### 3.2. Optimal Cryptophyte Prey for *M. rubrum* Growth

*M. rubrum* cultures showed different lag phase patterns in response to the different cryptophyte prey provided. The *M. rubrum* culture used as inoculum had been fed with *P. prolonga* before three weeks of starvation preceding the experiment. Our initial interpretation is that *M. rubrum* inoculum was still adapted to grow with its most recent *P. prolonga* prey. It has been shown that *M. rubrum* can grow with different species belonging to the TPG clade, and that old plastids are replaced when a new prey species is provided [44,45]. Plastid replacement from *T. amphioxeia* to *T. acuta* took approximately two weeks in an earlier study and occurred when *M. rubrum* was fed with only the other *Teleaulax* species [44]. Thus, after a period of adaptation *M. rubrum* plastids reflect those of the new prey [44,45], but the length of the adaptation period will vary with different cryptophyte prey provided. Therefore, the inoculum cells of *M. rubrum* probably had all their plastids replaced from *P. prolonga* when experiment 3 began. This would explain the better performance of *M. rubrum* cultures fed *P. prolonga* in the first two weeks while in the other cultures, *M. rubrum* specimens were progressively replacing their old *P. prolonga* plastids with those from the new cryptophyte prey provided. But after *M. rubrum* replaced its plastids with those from the new prey, there was a remarkable change of trends. Thus, cultures of *M. rubrum* with *P. prolonga* (CR10EHU), *T. minuta* (CR8EHU), and the *T. amphioxeia* strain (VGO1392) from the Galician Rías reached similar final yields on day 23. In the meantime, *M. rubrum* cultures fed *T. amphioxeia* (AND-A071) from the same region as *M. rubrum* continued a sustained exponential growth (µ = 0.18 d^−1^) for at least 12 more days, and reached a final yield 3-fold higher (up to 5 × 10^4^ cell mL^−1^) than the cell maxima attained with the other cryptophyte prey. These results agree with those reported by other authors who showed higher yields and growth rates for *M. rubrum* fed *T. amphioxeia* compared to other cryptophyte species [42,44].

It is worth highlighting that *M. rubrum* reached a much higher growth rate and final yield in cultures fed *T. amphioxeia* (AND-A070) from Huelva (southwest Spain) than with the same species, *T. amphioxeia* (VGO1392), with an identical partial plastid sequence, but from a different geographic area (Figure 8). The strain of *M. rubrum* (AND-A071) used in all the experiments was also isolated from Huelva. It has been claimed that *M. rubrum* exhibits genus-level but not species-level cryptophyte prey selection [44]. In the present work *M. rubrum* was grown with different species of *Teleaulax* and *Plagioselmis*, but best growth and yield were attained with the *T. amphioxeia* strain from the same location as the ciliate. It is possible that local adaptation allows a predator to recognize prey from the same geographical area. Alternatively, the two strains, despite having identical partial plastid sequences, may have other genetic differences that the southern strain of *M. rubrum* is able to recognize. 

Attempts to establish cultures of our local strains of *M. rubrum* and *M. major* from the Galician Rías to test these hypotheses have been unsuccessful. But we must note here that some of the densest *Dinophysis* cultures cited in the literature [22,24,47] are fed with *M. rubrum* and its *T. amphioxeia* prey isolated from the same locality as the dinoflagellate. The partial plastid 23S rDNA sequence from the Galician *Mesodinium* species (*M. rubrum* and *M. major*) coincides with that from field specimens of *Dinophysis*, and is 1 bp different from *T. amphioxeia* [45]. This sequence does not coincide with any other from the TPG cryptophytes known in the region. It is quite possible that we will not be able to establish successful cultures of our local strains of *Mesodinium* until we isolate a *Teleaulax*-like cryptophyte with the same partial plastid 23S rDNA sequence.

### 3.3. Best Results with Mass Production of *Dinophysis* and Other Considerations

Some of the best results so far attained with *D. acuminata* cultures in our laboratory, in terms of sustained exponential growth (3 weeks) and high yields, were obtained using *M. rubrum* fed *T. minuta*, with a very favorable (1:20) predator:prey ratio (Figure 6). This fact suggests that the lack of our own optimal cryptophyte prey may be to some extent compensated by using a high *M. rubrum*:*Dinophysis* ratio. Until now, most laboratory studies applied a D:M ratio of 1:10 [41,42,46]. However, in the experiments reported by these authors, *M. rubrum* was added to the cultures every three to 14 days, while in our experiments *M. rubrum* was all added the first day of the experiment.

To our knowledge, this is the first report of a *D. acuminata* culture in a photobioreactor. *Dinophysis acuminata* numbers increased 7-fold in 20 days (from 2 × 10^6^ to 13.8 × 10^6^). These are not very high values and they could have been improved had our production of *Mesodinium* been better at that moment. But results from earlier studies confirmed here have shown that a good (1:10) D:M ratio is a key factor to achieve high dinoflagellate yields [41,42]. There is limited literature regarding the distribution of *Dinophysis* cells through the culture vessel. In our study, *D. acuminata* cells were aggregated at the base of the small-scale culture flasks but were swimming in the water column forming patches in the photobioreactor. This response may reflect a difference in the availability of light between the two culture systems. By design, the photobioreactors are light limited at the base (Figure 7), which may have triggered a phototropic response of the cells, resulting in vertical migration towards the upper illuminated layers.

### 3.4. Variability in *Dinophysis* Cell Toxin Quota and Culture Strategies

This work was focused on the growth of two species of *Dinophysis* and *M*. *rubrum* in culture. However, often the purpose of high biomass cultures is to have a clean and reliable source of toxins needed to prepare standards for chemical analyses in monitoring programs. Earlier studies in the Swedish fjords and the Galician Rias showed changes of one order of magnitude in the toxin content of the same species throughout their growing season [48,49]. Maximal toxin per cell was usually found at the stationary phase, both in the field [48,49,50] and laboratory experiments [51,52], due to an imbalance between toxin production and reduced division. This imbalance resulted in an increased toxin per-cell (particulate) accumulation but also to higher levels of extracellular toxins. The latter could represent a very high percentage of the total amount of toxins produced by the cells in the field [50] and in laboratory experiments [51,52].

Values of toxin per cell observed under different experimental conditions, working with the same strains of *D. acuminata* and *D. acuta* (this work and other studies discussed below), also revealed a large variability (Table 1). In addition to the already cited imbalance between growth and toxin production, leading to the highest cell toxin quota, some other factors can be envisaged from the values depicted in Table 1. For example, prey-limited cells of *D. acuminata* and *D. acuta* had higher toxin per cell than the parallel treatment with well-fed cells in experiments detailed by Portela et al. [34]. Lack of food (or the excess of it) has been already highlighted by other authors as a key factor promoting fast (well fed) or reduced (prey-limited) division [41,46]. Another striking observation is the high values of toxin per cell in well illuminated cultures versus those in low light conditions (Table 1). In that case, light seems to have had a strong and direct positive effect on toxin production. This effect would act presumably through the enhancement of photosynthetic activity required to generate reduction power to synthesize secondary metabolites (i.e., toxins) [41,47]. Some of the lowest values correspond to cells that were grown at the maximal temperature (19 °C) and light hours (16L:8D cycle) in experiment 3 of this work. These conditions favored a maximal division rate in *D. acuminata* and *D. acuta* cultures that were harvested for toxins extraction on day 6, during early exponential growth. It is well known that higher temperature (within a species-specific range) and hours of light promote higher division rates in *Dinophysis* cultures (33,41,47). Increased division “dilutes” the cell toxin quota. In other words, there is a negative correlation between division and toxin accumulation rates. The origin of the *Mesodinium* prey, i.e., a *M. rubrum* strain from Denmark versus the strain from southwest Spain used in this work, was also found to have an effect on *Dinophysis* growth and toxin accumulation [34,53].

Some extremely high values of cell toxin quota were observed in cultures growing in suboptimal conditions and with a very low division rate. That was the case with *D. acuta* fed a Danish strain of *M. rubrum* [34]. The record values of total toxin (particulate + dissolved, marked with an * in Table 1) per cell were observed in some mass cultures of *D. acuminata* grown for toxins sourcing and harvested with DIAON® adsorbing resins (Table 1 in bold). They corresponded to a slow growing, low-density (320 cells L^−1^) culture of *D. acuminata* that was harvested at the stationary phase when nanoflagellate contamination was detected. Values of toxin per cell estimated when total toxins (harvested with resins) are measured are misleading. The dissolved toxins detected have been accumulated from the toxins released by cells growing in the preceding exponential phase of the culture, and which may have already died and contributed to the dissolved toxins pool. In these cases, it is more appropriate to express toxin content per unit of culture volume. 

The development of passive samplers for in situ detection of lipophilic toxins with “solid-phase adsorption toxin tracking” (SPATT) resins provided a valuable new tool for the toxin dynamic studies [54]. Before that, extracellular toxins released by the cells in the water were not quantified. There is controversy on the advantages of the SPATT resins for early warning of *Dinophysis* blooms, but their value for research on physiology and toxin production dynamics is unquestionable [50]. The predominance of dissolved versus particulate toxins, detected with SPATT resins, has been reported in the stationary phase during blooms of *D. acuta* in New Zealand [54] and in laboratory experiments with the same species [52]. This observation led to the deployment of in situ toxin-harvesting devices as an alternative to cultures for toxins sourcing [55]. 

All the above observations give hints on the appropriate strategies to follow in order to get high numbers of toxic cells. *Dinophysis* cultures can be produced following two stages, with a different set of conditions promoting either growth or toxin accumulation. The first “production stage”, will aim to reach the maximal cell density (yield) through good division rates. This will be supported by a high temperature (≥19 °C), favorable D:M ratio (20:1) using the preferred prey, and optimal light intensity according to each species/strain of *Dinophysis*. The second “seasoning stage”, will aim to reach maximal values of toxin per cell and extracellular toxins This situation will be triggered via *Dinophysis* starvation, lowering the temperature and any additional factor contributing to an arrest of cellular division, i.e., forcing the imbalance between division and toxin production rates in favor of the latter. 

## 4. Conclusions

*Dinophysis acuminata* and *D. acuta* exhibited higher growth rates when grown in K(-Si) medium, likely reflecting the presence of ammonia which is the preferred N source. *M. rubrum* showed a strain-specific growth response to the cryptophyte prey supplied: enhanced growth with *T. amphioxeia* isolated from the same geographic area (Huelva, southwest Spain) as compared with the same species from the Galician Rías (northwest Spain). Maximal growth rates in *D. acuminata* and *D. acuta* cultures were achieved with *M. rubrum* fed *T. amphioxeia* from the same region, therefore “what is better for *M. rubrum* is better for *Dinophysis*”. The use of diluted L1 and f/2 media can be helpful for maintenance of *M. rubrum* and cryptophytes by keeping excessive cryptophyte growth and undesirable contaminants at bay. A favorable (1:20) D:M ratio, the key factor to high division rates, combined with the use of K(-Si) medium, may alleviate the lack of the optimal local cryptophye strain (of the *Teleaulax*/*Plagioselmis*/*Geminigera* clade), to produce mass cultures of *Dinophysis*. Galician *Mesodinium* and *Dinophysis* partial plastid 23S rDNA sequences differ by just one nucleotide from those in southern Spain specimens. This difference seems to suggest some degree of variability between those organisms affecting the growth of the southern *Mesodinium* with the northern cryptophyte prey. The lack of cultures of local strains of *Teleaulax*-like cryptophytes with the same partial 23S rDNA sequence could also explain unsuccesful attempts to establish cultures of the local *Mesodinium* species (*M. rubrum* and *M. major*) in the Galician Rías with the southern strains of *T. amphioxeia*. Practical recommendations for mass production of *Dinophysis* with high toxin content are given.

## 5. Materials and Methods 

### 5.1. Cultures, Culturing Conditions, and Single-Cell Isolated Field Specimens

*Dinophysis* cultures were established from water samples from the Galician Rías Baixas (northwest Spain). *Dinophysis acuminata* (strain VGO1391) was isolated from Ría de Vigo in July 2016 and *D. acuta* (VGO1065) from Ría de Pontevedra in October 2010, both rías being part of the Galician Rías Baixas (northwest Spain). The ciliate *M. rubrum* (AND-A071) was isolated in 2007 from samples collected off Huelva (southwest Spain). Cryptophytes used in the culture experiments were from three different regions in Spain. *Teleaulax amphioxeia* (AND-A070) was isolated from samples off Huelva in 2007; another strain of *T. amphioxeia* (VGO1392) was isolated from Ría de Vigo (northwest Spain) in 2017, and the cryptophyte strains *Plagioselmis prolonga* (CR10EHU), *Teleaulax gracilis* (CR6EHU), and *Teleaulax minuta* (CR8EHU) from the Nervión River estuary, Bay of Biscay (north Spain). These cryptophytes have been found to be eaten by *M. rubrum* and plastid replacement in the ciliate with those of the new prey, demonstrated with partial sequencing of their 23S rDNA [45]. All cultures were grown with diluted (1:20) f/2 [29] or L1 medium [30] culture media prepared with autoclaved seawater at pH 8.00 ± 0.02 and salinity of 32 psu. They were kept in a temperature controlled room at 15 ± 1 °C and provided ~150 μmol photons m^2^ s^−1^ PAR (photosynthetically active radiation) on a 12 h light:12 h dark cycle. Irradiance was delivered by Osram LED 30W-cold light, 6400 °K, tubes (OSRAM GmbH, Munich, Germany). All cultures were non-axenic.

**Figure 9:**
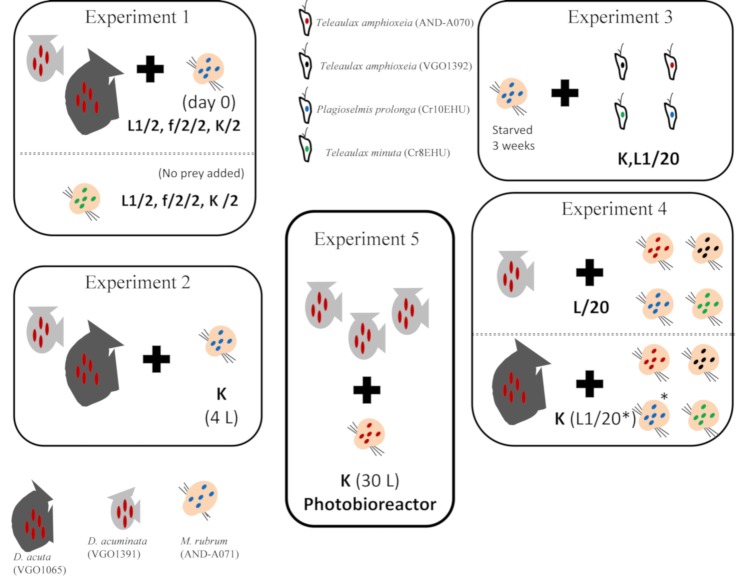
Graphic summary of the species/strains used in the experiments and trophic interactions investigated. Experiment 1: Phototrophic growth of *D. acuminata*, *D. acuta*, and *M*. *rubrum* with different culture media. Experiment 2: Scaling up mixotrophic cultures of *D. acuminata* and *D. acuta* with K medium. Experiment 3: Mixotrophic growth of *M. rubrum* with different cryptophytes. Experiment 4. Optimal cryptophyte prey for *M. rubrum* and best *M. rubrum* ratio to feed *Dinophysis*. All media were Si free and diluted (1:2). * indicates one treatment using L1 medium with a 1:20 dilution.

A second species of *Mesodinium*, *M*. *major*, common in Galician coastal waters during blooms of *Dinophysis*, was considered in this study. Attempts to cultivate local strains of the two species of *Mesodinium*, *M*. *rubrum* and *M. major*, have been unsuccessful. Field specimens of *M*. *rubrum* and *M. major* were isolated from water samples from the Galician Rías for partial sequencing of their plastid gene 23S rDNA to compare it with those from cultivated *M. rubrum* (AND-A071), and with the local cultivated strains of *D. acuminata* (VGO1391), *D. acuta* (VGO1065), and *T. amphioxeia* (VGO1392). Cells were picked manually, one by one, with a capillary pipette under a Zeiss Invertoscop D (Karl Zeiss, Jena, Germany) microscope, washed in 3 drops of sterile distilled water and transferred to PCR tubes (see Section 5.8). Species identification of *Dinophysis* and *Mesodinium* species was based on morphological characteristics observed by light microscopy. A graphic diagram with the names of the species used in different experiments and their trophic interactions is shown in Figure 9.

### 5.2. Cell Counts and Growth Rate Estimates

To estimate cell densities, specimens in 2 mL subsamples from 3 aliquots were fixed with acidic Lugol’s solution (0.5%) and counted. *Dinophysis* species and *M. rubrum* were counted in a 1 mL Sedgwick-Rafter (Pyser-SGI S50, Pyser Optics, Kents, UK) counting chamber with a Zeiss Invertoscope D microscope at 100× or 250× magnification. Cryptophyte species were counted either in a 1 mL Sedgwick-Rafter chamber or in a Neubauer-type hemocytometer (depending on the cell density) at 200×.

Specific growth rates (μ) were calculated from

μ = (ln N_2_ − ln N_1_/t_2_ − t_1_)

where N_1_ and N_2_ denote cell numbers (cell mL^−1^) recorded at time t_1_ and t_2_ (days), respectively.

A one-way ANOVA was used to identify significant differences in cell densities among treatments. Values of *p* < 0.05 were considered statistically significant. Statistical analyses were carried out with the RStudio, version 3.3.2, (RStudio, Boston, MA, USA).

### 5.3. Experiment 1. Phototrophic Growth of *D. acuminata*, *D. acuta*, and *M. rubrum* with Different Culture Media

Culture experiments were set up to compare phototrophic growth of *D. acuminata* and *D. acuta* grown in autoclaved seawater enriched with diluted (1:2) L1(-Si) [30], f/2(-Si) [29], and K(-Si) [36] culture media. To observe phototrophic growth of *Dinophysis*, without interferences from mixotrophic feeding, *M. rubrum,* previously fed *P. prolonga*, was added as prey only on day 0. Initial *Dinophysis* (D) cell concentrations were adjusted to approximately 150 and 200 cells mL^−1^ for *D. acuminata* and *D. acuta* respectively and *M. rubrum* (M) concentrations were adjusted to have a 1:10 D:M ratio. 

To observe phototrophic growth of *M. rubrum,* cultures of the ciliate fed *T. minuta,* were deprived of prey for 3 weeks and the absence of cryptophyte cells confirmed by light microscopy observations. Thereafter, an experiment was run to compare phototrophic growth of *M. rubrum* in autoclaved seawater enriched with L1(-Si), f/2(-Si), and K(-Si) media. Experiments were carried out in triplicate in 250 mL Erlenmeyer flasks and the same conditions described in 5.1. Samples were collected every 2 d except in the case of the experiment with *D. acuta* (once a week) due to the already known very slow growth of this species when prey is not added [33].

### 5.4. Experiment 2. Scaling up Mixotrophic Cultures of *D. acuminata* and *D. acuta* Cultures with K(-Si) Medium

*Dinophysis acuminata* and *D. acuta* cultures volume was scaled-up from 100 mL to medium-scale volume (4 L) cultures. Mixotrophic cultures of *D. acuminata* and *D. acuta* were carried out three times in triplicate 4 L flasks with K(-Si) medium at 19 °C and provided 150–200 μmol photons m^2^ s^−1^ PAR on a 16 h L:8 h D cycle. *Dinophysis* and *M. rubrum* cells, grown in autoclaved seawater enriched with K(-Si) medium, were previously acclimated to the culture parameters. The initial *Dinophysis* cell concentrations were adjusted to 200 cells mL^−1^ and the D:M ratio to 1:10 and then adjusted to 1:5 every 2 days. 

Therefore, *D. acuminata* and *D. acuta* culture volumes were scaled-up periodically with *M. rubrum* grown with the cryptophyte *P. prolonga.* Samples were taken every day. On day 6, cultures were filtered through 25 mm GF/D glass microfiber filters (Cole-Parmer Instrument, Filter-Lab, Vernon, IL, USA), the filter with the filtered material placed in 15 mL centrifuge tubes and filled with MeOH (analytical grade) and kept in the deep-freeze at −20 °C until extraction for liquid chromatography coupled to tandem mass spectrometry (LC–MS/MS) analysis (see Section 5.10 and Section 5.11).

### 5.5. Experiment 3. Mixotrophic Growth of *M. rubrum* with Different Cryptophytes

Mixotrophic growth of *M. rubrum* fed different cryptophyte species was studied. *M. rubrum* fed *P. prolonga* with K(-Si) medium was starved for 3 weeks and the absence of cryptophyte cells was confirmed with the light microscope before the experiment. Thereafter, three cryptophyte species, *T. amphioxeia* (strains AND-A070 and VGO1392), *T. minuta* (CR8EHU), and *P. prolonga* (CR10EHU), were given on day 0 to *M. rubrum* grown with K(-Si) medium to identify the optimal prey for the ciliate. The initial *M. rubrum* cell concentrations were adjusted to 10^3^ cells mL^−1^ and a *M. rubrum*:*cryptophyte* (M:C) ratio of 1:10. Cultures of *M. rubrum* with *P. prolonga* and diluted (1:20) L1(-Si) medium were used as an internal control. All cultures were carried out in triplicate 250 mL Erlenmeyer flasks and the same conditions described in Section 5.1. Samples were taken every 2 days.

### 5.6. Experiment 4. Optimal Cryptophyte Prey for *M. rubrum* and Best *M. rubrum* Ratio to Feed *Dinophysis*

The next step was to investigate if the optimal prey for *M. rubrum* was also the best to feed *Dinophysis*. *M. rubrum* cultures, each one grown with different cryptophyte species (*T. amphioxeia,* AND-A070 and VGO1392; *T. minuta*, CR8EHU; *T. gracilis*, CR6EHU; and *P. prolonga,* CR10EHU) were provided as prey to *D. acuminata*, grown with L1/20(-Si) medium. Likewise, *M. rubrum* fed *T. amphioxeia* (AND-A070 and VGO1392), and *P. prolonga* (CR10EHU) was given to *D. acuta* (grown with K(-Si) and L1/20(-Si) medium) at day 0, to determine the optimal cryptophyte prey for *M. rubrum* to be used as prey for this species. The initial *Dinophysis* cell concentrations were adjusted to 150 cells mL^−1^ and the D:M ratio was 1:10. In addition, two culture experiments were carried out to compare *D. acuminata* mixotrophic growth in autoclaved seawater enriched with K(-Si) medium with *M. rubrum*, fed *T. minuta* (CR8EHU), added as prey only on day 0 and D:M ratios adjusted to 1:10 and 1:20 respectively. Cultures were carried out in triplicate 250 mL Erlenmeyer flasks and the same conditions described in Section 5.1. Samples were taken every 2 days.

### 5.7. Experiment 5. Mass Production of *D. acuminata* in 30 L Photobioreactors

Mixotrophic growth of *D. acuminata* in large volumes was studied in a photobioreactor. This photobioreactor, model AIS1316 from Aqualgae (Aqualgae S.L., A Coruña, Spain), has a polymethyl metacrylate (PPM), 250 mm diameter, and 30 L column supported on a stainless steel structure. Light is provided by 3 vertical LED tubes (cold light, 6400 °K); light intensity, photoperiod, temperature, and pH are controlled by an automatic mini-pic sensor (Siemens AG, Munich, Germany). Cultures in the photobioreactor were initiated with a volume of 12.5 L and a density of 160 cells mL^−1^ of *D. acuminata* in K(-Si) medium. No aeration was provided. *Dinophysis* (D) were fed *M. rubrum* (M) grown with *T. amphioxeia (*AND-A070) at a D:M ratio 1:1, 2−3 times a week. This ratio was readjusted to 1:5 D:M when a density of 500 cells mL^−1^ of *Dinophysis* was reached. Then it was adjusted to a 1:5 (D:M) ratio. Aliquots for cell counts were taken with a 5 mL pipette at the center of the water column after gentle circular agitation of the bioreactor. When the experiment finished, particulate and dissolved toxins were collected from the bioreactor with DIAON resins (see Section 5.10).

### 5.8. DNA Extraction, PCR Amplification and Sequencing 

Field specimens isolated by micromanipulation (see Section 5.1) of *D. acuminata*, *D. acuta*, *M. rubrum*, and *M. cf. major* from the Galician Rías were transferred to 200 μL PCR tubes and kept at −20 °C for 24 h before direct amplification. For DNA extraction of species already in culture, 1 mL of each cryptophyte species used in the experiments, and of *M. rubrum* (AND-A071) were centrifuged for 5 min at ×12,000 *g* in a mini Spin Eppendorf centrifuge (Eppendorf AG, Hamburg, Germany), pellets rinsed in MilliQ water, centrifuged again, and then DNA was extracted using Chelex^®^ 100 (Bio-Rad, Hercules, CA, USA) following the extraction procedure of Richlen & Barber [56]. For amplification of partial plastid 23S rDNA sequences, universal primers p23Sr_f1 (5′-GGA CAG AAA GAC CCT ATG AA-3′), and 23Sr_r1 (5′-TCA GCC TGT TAT CCC TAG AG-3′) [57] were used. The PCR reactions were performed using a thermocycler (Eppendorf AG, Hamburg, Germany), following the conditions detailed by these authors. PCR reaction mixtures (25 μL) contained 1 to 3 *Dinophysis* cells each, 1 mM MgCl_2_, 2.5 μL 10× PCR buffer, 125 nM of each primer, 25 nM dNTPs, and 0.65 units Taq DNA polymerase (Bioline Reagents Ltd., London, UK). The PCR products were analyzed by 1.5% agarose gel electrophoresis. The amplified products were purified using an ExoSAP-IT (USB Corporation, Cleveland, OH, USA). Finally, the PCR products obtained were sequenced using the ABI PRISM BigDye Terminator Cycle Sequencing Ready Reaction Kit and an Applied Biosystems ABI 310 automated sequencer (CACTI, University of Vigo, Vigo, Pontevedra, Spain).

### 5.9. Phylogenetic Analysis

The partial sequences of plastid 23S rDNA (373bp) were aligned using CLUSTAL W [58] in Bioedit [59]. Phylogenetic analyses of 23S rDNA were performed using Tamura-Nei model [60]. Evolutionary analyses were conducted in MEGA 7, version 7.0. (Microsoft Windows applications, graphical user interface) [61]. Maximum likelihood (ML) phylogenetic analyses were conducted. The phylogenetic tree was represented using the ML method with bootstrap values (*n* = 1000). The tree with the highest log likelihood (−639.0779) was shown. The percentage of trees in which the associated taxa clustered together was shown next to the branches. Initial tree(s) for the heuristic search were obtained automatically by applying Neighbor-Joining and BioNJ algorithms to a matrix of pairwise distances estimated using the Maximum Composite Likelihood (MCL) approach, and then selecting the topology with superior log likelihood value. The tree was drawn to scale, with branch lengths measured in the number of substitutions per site. The analysis involved 17 sequences. Codon positions included were 1st + 2nd + 3rd + Noncoding. All positions containing gaps and missing data were eliminated.

### 5.10. Harvesting and Total Toxin Extraction from *Dinophysis* Cultures

Both particulate and extracellular toxins released in the culture medium from mass cultures produced to extract and biorefine toxins were harvested with polyaromatic adsorbent resin Diaion™ HP-20SS resin, Ø 75–150 µm SUPELCO (Bellefonte, PA, USA). First, *Dinophysis* cells were lysed by addition of acetone (final concentration 7%). Then the Diaion™ HP-20SS resin had to be activated before use, as described in MacKenzie et al. [54] and applied by Pizarro et al. [49,50]. In short, batches of adsorbent resin were washed several times with at least 10 volumes (10 solvent: 1 resin) of MeOH, to remove fines and leachable material; then, hydrated by soaking in MilliQ water, and drained through a 95 mm mesh sieve. Activated resin (2 g HP-2055 per L of culture) was added to the lysed-cells culture and stirred with a magnetic bar at low speed, very gently, for 24 h to ensure resuspension of the particles in the water column. After incubation, the resin retained by filtration over a mesh (20 µm), thoroughly rinsed with MilliQ water to remove salts from the culture medium, was transferred to a glass Petri dish. This was dried in an oven (3 h, 50 °C) and then kept at −20 °C until analysis.

### 5.11. Toxin Analyses

Toxin analyses were carried out at the Marine Institute in Galway, Ireland. The resin was transferred into a glass beaker and extracted by sonication with MeOH for 1 h. The extract was filtered through a SPE cartridge (empty with frit) and transferred into a volumetric flask. The remaining resin was further sonicated in MeOH several times until LC–MS/MS indicated that >95% of the toxin was extracted, with each extract decanted into the same volumetric flask which was then made up to volume with MeOH. Samples were filtered through a plugged (with cotton wool) glass pipette into HPLC vials for analysis. Next, they were hydrolyzed (to convert any OA group esters back to the parent compounds) by adding 125 µL 2.5 M NaOH to 1 mL of sample, placed in a water bath set at 76 °C for 10 min, cooled and then neutralized with 2.5 M HCl. Both the unhydrolyzed and the hydrolyzed samples were analyzed by LC–MS/MS to determine the level of esters present in the samples.

LC–MS/MS analysis of the resin extracts was carried out with a Waters Acquity UPLC system coupled to a Xevo G2-S QToF monitoring in MS^e^ mode in both positive and negative modes (*m*/*z* 100−1200), using leucine enkephalin as the reference compound. The cone voltage was 40 V, collision energy was 50 V, the cone and desolvation gas flows were set at 100 and 1000 L/h, respectively, and the source temperature was 120 °C. Analytical separation was performed on an Acquity UPLC BEH C18 (50 × 2.1 mm, 1.7 µm) column (Waters, Wexford, Ireland). Binary gradient elution was used, with phase A consisting of H_2_O and phase B of CH_3_CN (95%) in H_2_O (both containing 2 mM ammonium formate and 50 mM formic acid). The injection volume was 2 µL and the column and sample temperatures were 25 °C and 6 °C, respectively. 

In positive mode the gradient was from 30% to 90% B over 5 min at 0.3 mL/min, held for 0.5 min, and returned to the initial conditions and held for 1 min to equilibrate the system. Processing of results was performed using Waters Targetlynx software pulling out the masses for PTX2 (*m*/*z* 876.51 + 881.46). In negative mode the gradient was from 5% to 90% B over 2 min at 0.3 mL/min, held for 1 min, and returned to the initial conditions and held for 1 min to equilibrate the system. Processing of results was performed using Waters Targetlynx software pulling out the mass for OA and DTX2 (*m*/*z* 803.45). PTX2, OA and DTX2 were quantitated using certified reference materials from the National Research Council, Canada.

## Figures and Tables

**Table 1:** Intracellular (particulate, pg cell^−1^) and total toxin (particulate + dissolved, pg mL^−1^cell^−1^, marked with *) contents in cultures of *D. acuta* (VGO1065) and *D. acuminata* (VGO1391) from this work and from other experiments carried out with the same strains isolated in Vigo. Abbreviations: Ref. = reference; ES = *M. rubrum* strain from Huelva, Spain, or DK = from Denmark, V = volume, T = temperature; sta = stationary phase; exp = exponential phase; L:D = light:dark; OA = okadaic acid; DTX2 = dinophysistoxin 2; PTX2 = pectenotoxin 2.

Species	Ref.	Experimental Conditions	V (mL)	T (°C)	L:D Cycle (h)	Medium	OA (pg cell^−1^)	DTX2 (pg cell^−1^)	PTX2 (pg cell^−1^)
*D. acuta*	[34]	Well-fed (ES)	250	15	12:12	L1-Si/20	12.2 ± 2.3	4.4 ± 0.9	22.2 ± 9.4
	[34]	Well-fed (ES)	150	17	14:10	L1-Si/20	41.0 ± 4.9	17.4 ± 4.1	38.0 ± 8.2
		Prey-limited (ES)	150	17	14:10		74.1 ± 8.2	32.4 ± 3.8	59.3 ± 11.8
	[34]	Well-fed (DK)	150	17	14:10	L1-Si/20	35.9 ± 7.07	16.5 ± 0.8	70.0 ± 0.8
		Prey-limited (DK)	150	17	14:10		38.6 ± 4.5	19.0 ± 2.3	43.6 ± 6.2
	[33]	Low light	250	15	12:12	L1-Si/40	3.3 ± 1.6	2.2 ± 0.1	71.5 ± 14.2
		High light	250	15	12:12		50.2 ± 20.1	35.4 ± 17.4	187.8 ± 104.1
	This work	Mass culture-sta	1450	15	12:12	L1-Si/20	30.2 *	7.3 *	48.2 *
		Mass culture-sta	3500	15	12:12	K-Si	15.5 *	5.2 *	50.5 *
		Mass culture-exp	4000	19	16:8	K-Si	7.7	2.9	8.2
		Mass culture-sta	5000	15	12:12	L1-Si/20	61.5 *	20.3 *	3 *
*D. acuminata*	[34]	Well-fed (ES)	250	15	12:12	L1-Si/20	35.2 ± 6.8		
	[34]	Well-fed (ES)	150	17	14:10	L1-Si/20	6.0 ± 2.8		
		Prey-limited (ES)	150	17	14:10		21.5 ± 0.7		
	[34]	Well-fed (DK)	150	17	14:10	L1-Si/20	9.8 ± 1.7		
		Prey-limited (DK)	150	17	14:10		32.3 ±4.7		
	[33]	Low light	250	15	12:12	L1-Si/40	14.7 ± 12.1		
		High light	250	15	12:12		41.4 ± 4		
	This work	Mass culture-sta	2200	15	12:12	L1-Si/20	33.3 *		
		Mass culture-sta	2700	15	12:12	L1-Si/20	122.2 *		
		Mass culture-exp	4000	19	16:8	K-Si	9.9		
		Mass culture-sta	4500	15	12:12	K-Si	20.3 *		
		Mass culture-sta	17,900	15	12:12	K-Si	28.9 *		

**Table 2:**
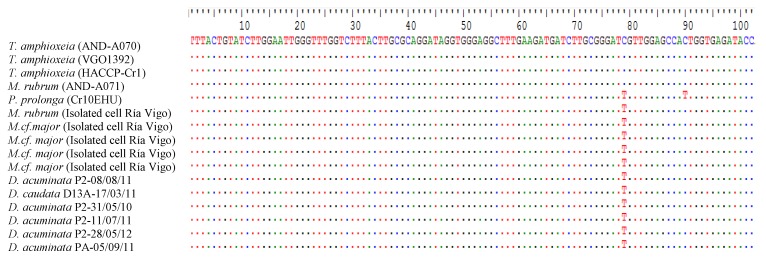
Alignment of the first 100 bp from the partial (373 bp) plastid 23S rDNA of *T. amphioxeia* (strains from Huelva and Vigo) and *P. prolonga* (Basque Country, north Spain) cultures, *M. rubrum* (cultivated strains from Huelva and isolated cells from Vigo), *M. major*, and *Dinophysis* cells isolated from water samples collected in Ría de Vigo and Ria de Pontevedra (Galician Rias Baixas, northwest Spain). The whole 373 bp partial plastid sequence was identical except in positions 79 and 90 shown here. These correspond to positions 2123 and 2134 in the whole plastid 23S rRNA gene (referred to *Rhodomonas salina,* NCBI Reference Sequence: NC_009573.1).
